# *catena*-Poly[[nickel(II)-μ-1,3-dimethyl-2,6-dioxo-7*H*-purinato-κ^2^ *N* ^7^:*N* ^9^] hydroxide]

**DOI:** 10.1107/S160053680800737X

**Published:** 2008-03-29

**Authors:** Lin-Heng Wei

**Affiliations:** a College of Environment and Planning, Henan University, Kaifeng 475001, People’s Republic of China

## Abstract

The title complex, {[Ni(C_7_H_7_N_4_O_2_)]OH}_*n*_, has been prepared through hydro­thermal synthesis. The asymmetric unit contains one [Ni(TH)]^+^ cation (TH is the theophylline anion) and one hydroxide anion. The Ni^2+^ ion is coordinated by two N atoms from two neighboring theophylline anions. The alternating linkage of the Ni^2+^ cation and theophylline anion results in a one-dimensional chain along the [010] direction. Intermolec­ular O—H⋯O hydrogen bonds are present n the crystal structure.

## Related literature

For related literature, see: Horikoshi & Mochida (2006 ▶); Robin & Fromm (2003 ▶).
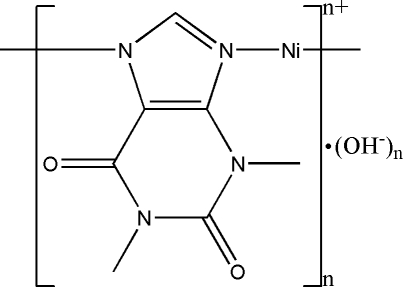


## Experimental

### 

#### Crystal data

[Ni(C_7_H_7_N_4_O_2_)]OH
*M* *_r_* = 254.88
Monoclinic, 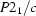

*a* = 11.399 (3) Å
*b* = 11.533 (2) Å
*c* = 6.9807 (15) Å
β = 101.993 (3)°
*V* = 897.7 (3) Å^3^
*Z* = 4
Mo *K*α radiation
μ = 2.15 mm^−1^
*T* = 298 (2) K
0.48 × 0.24 × 0.08 mm

#### Data collection

Bruker SMART APEX CCD area-detector diffractometer
Absorption correction: multi-scan (*SADABS*; Sheldrick, 2001 ▶) *T*_min_ = 0.425, *T*_max_ = 0.847
4701 measured reflections
1753 independent reflections
1592 reflections with *I* > 2σ(*I*)
*R*_int_ = 0.024

#### Refinement

*R*[*F* ^2^ > 2σ(*F* ^2^)] = 0.028
*wR*(*F* ^2^) = 0.078
*S* = 1.07
1753 reflections
142 parameters
7 restraints
H atoms treated by a mixture of independent and constrained refinement
Δρ_max_ = 0.36 e Å^−3^
Δρ_min_ = −0.43 e Å^−3^

### 

Data collection: *SMART* (Bruker, 2001 ▶); cell refinement: *SAINT-Plus* (Bruker, 2001 ▶); data reduction: *SAINT-Plus*; program(s) used to solve structure: *SHELXS97* (Sheldrick, 2008 ▶); program(s) used to refine structure: *SHELXL97* (Sheldrick, 2008 ▶); molecular graphics: *PLATON* (Spek, 2003 ▶); software used to prepare material for publication: *PLATON*.

## Supplementary Material

Crystal structure: contains datablocks global, I. DOI: 10.1107/S160053680800737X/at2551sup1.cif

Structure factors: contains datablocks I. DOI: 10.1107/S160053680800737X/at2551Isup2.hkl

Additional supplementary materials:  crystallographic information; 3D view; checkCIF report

## Figures and Tables

**Table 1:** Hydrogen-bond geometry (Å, °)

*D*—H⋯*A*	*D*—H	H⋯*A*	*D*⋯*A*	*D*—H⋯*A*
O3—H3⋯O1^i^	0.832 (11)	2.023 (12)	2.851 (3)	173 (5)

Symmetry code: (i) 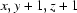.

**Fig. 1.**
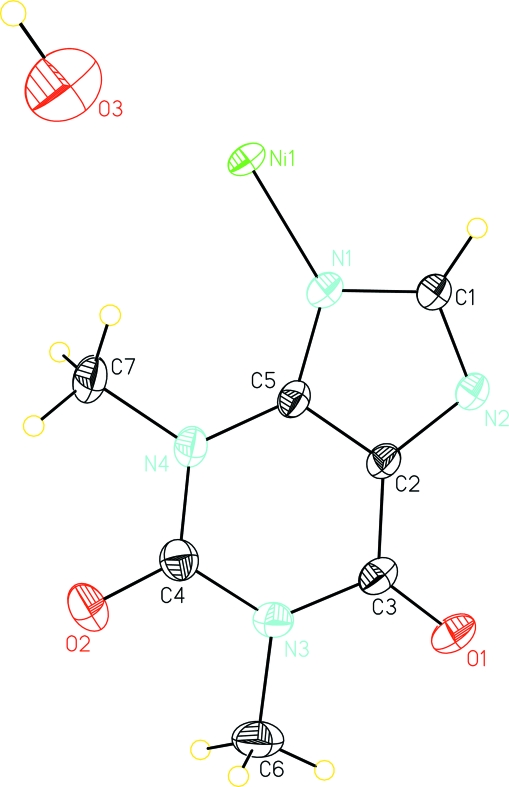
Asymmetry structural unit of (I). Displacement ellipsoids for non-H atoms are drawn at the 30% probability level.

**Fig. 2.**
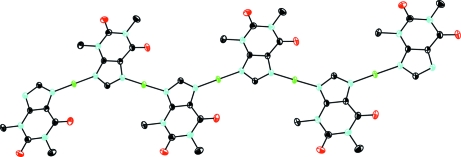
One-dimensional chain structure of the cations {[Ni(TH)]+}n. Hydrogen atoms are omitted for clarity.

## supplementary crystallographic information

### Comment

The rational design, synthesis and characterization of coordination polymers construct from transition metal ions, especially the first-row transition metal, and various organic ligands linked with covalent bonds have still been actively researched as one of highly topical research areas aiming to obtain fascinating structures as well as special properties such as magnetism, catalysis, molecular recognition, ion exchange, nonlinear optical behavior and electrical conductivity (Robin & Fromm, 2003; Horikoshi & Mochida, 2006). Herein we present a one-dimensional,linear transition metal complexes, namely {[Ni(TH)]OH}_n_(TH = theophylline anion), (I).

Each asymmetry unit of the title compound (I) consists of one [Ni(TH)]^+^ cation and one isolated hydroxyl anion (Fig.1). Ni^2+^ adopts a two-coordinate coordination mode and coordinated by two nitrogen atoms from two neighboring theophylline anions with average Ni—N length 1.861° and N—Ni—N angle 177.25° (Table 1), respectively. The short Ni—N distances in the compound are caused by the low coordination numbers and highly positive charges. The alternate linkers of Ni^2+^ ion and theophylline anion within which two adjacent anions are in the *trans*-position finally give rise to a one-dimensional chain (Fig.2). To best of our knowledge, the title complex is firstly reported. We found 3,5-dinitrobenzoic acid takes an key role in controlling the formation of the title compound. If 3,5-Ddinitrobenzoic acid was not added into the reaction system, the compound can't be obtained. Moreover, we also found basic medium NaOH must be added into the reaction system. Otherwise these compounds can't be prepared. We think that 3,5-dinitrobenzoic acid here acts as a reaction template. Additionally, the effect of the basic medium (NaOH) made NH group of theophylline deprotonate leading to the formation of a monoanionic bidentate ligand.

### Experimental

A mixture of NiCl_2_.6H_2_O (0.50 mmol, 0.12 g), 3,5-dinitrobenzoic acid (0.50 mmol, 0.110 g), theophylline monohydrate (0.50 mmol, 0.09 g), NaOH (0.5 mmol, 0.02 g) and H_2_O (20 ml) in the mole ratio 1:1:1:1:2 were heated in a Teflon-lined steel autoclave inside a programmable electric furnace at 1433 K for 72 h. After cooling the autoclave to room temperature for 36 h, brown crystals suitable for single-crystal X-ray diffraction were obtained.

### Refinement

H atoms bonded to O atom were located from the difference maps and refined with distance restraints O—H = 0.82 (1) Å. All the remaining H atoms were positioned geometrically, with C—H = 0.93–0.96 Å, and refined as riding, with *U*_iso_(H) = 1.2*U*_eq_(aromatic C) or 1.5*U*_eq_(methyl C).

### Crystal data

**Table:**

[Ni(C_7_H_7_N_4_O_2_)]OH	*F*_000_ = 520
*M**_r_* = 254.88	*D*_x_ = 1.886 Mg m^−^^3^
Monoclinic, *P*2_1_/*c*	Mo *K*α radiation λ = 0.71073 Å
Hall symbol: -P 2ybc	Cell parameters from 1720 reflections
*a* = 11.399 (3) Å	θ = 2.2–28.0º
*b* = 11.533 (2) Å	µ = 2.15 mm^−^^1^
*c* = 6.9807 (15) Å	*T* = 298 (2) K
β = 101.993 (3)º	Block, brown
*V* = 897.7 (3) Å^3^	0.48 × 0.24 × 0.08 mm
*Z* = 4	

### Data collection

**Table:**

Bruker SMART APEX CCD area-detector diffractometer	1753 independent reflections
Radiation source: fine-focus sealed tube	1592 reflections with *I* > 2σ(*I*)
Monochromator: graphite	*R*_int_ = 0.024
*T* = 298(2) K	θ_max_ = 26.0º
ω scans	θ_min_ = 1.8º
Absorption correction: multi-scan(SADABS; Sheldrick, 2001)	*h* = −14→5
*T*_min_ = 0.425, *T*_max_ = 0.847	*k* = −13→14
4701 measured reflections	*l* = −8→8

### Refinement

**Table:**

Refinement on *F*^2^	Secondary atom site location: difference Fourier map
Least-squares matrix: full	Hydrogen site location: inferred from neighbouring sites
*R*[*F*^2^ > 2σ(*F*^2^)] = 0.028	H atoms treated by a mixture of independent and constrained refinement
*wR*(*F*^2^) = 0.078	*w* = 1/[σ^2^(*F*_o_^2^) + (0.048*P*)^2^ + 0.1385*P*] where *P* = (*F*_o_^2^ + 2*F*_c_^2^)/3
*S* = 1.07	(Δ/σ)_max_ = 0.001
1753 reflections	Δρ_max_ = 0.36 e Å^−^^3^
142 parameters	Δρ_min_ = −0.43 e Å^−^^3^
7 restraints	Extinction correction: none
Primary atom site location: structure-invariant direct methods	

### Special details

**Table:**

Geometry. All e.s.d.'s (except the e.s.d. in the dihedral angle between two l.s. planes) are estimated using the full covariance matrix. The cell e.s.d.'s are taken into account individually in the estimation of e.s.d.'s in distances, angles and torsion angles; correlations between e.s.d.'s in cell parameters are only used when they are defined by crystal symmetry. An approximate (isotropic) treatment of cell e.s.d.'s is used for estimating e.s.d.'s involving l.s. planes.
Refinement. Refinement of *F*^2^ against ALL reflections. The weighted *R*-factor *wR* and goodness of fit *S* are based on *F*^2^, conventional *R*-factors *R* are based on *F*, with *F* set to zero for negative *F*^2^. The threshold expression of *F*^2^ > σ(*F*^2^) is used only for calculating *R*-factors(gt) *etc*. and is not relevant to the choice of reflections for refinement. *R*-factors based on *F*^2^ are statistically about twice as large as those based on *F*, and *R*- factors based on ALL data will be even larger.

### Fractional atomic coordinates and isotropic or equivalent isotropic displacement parameters (Å^2^)

**Table:**

	*x*	*y*	*z*	*U*_iso_*/*U*_eq_	
Ni1	0.48633 (3)	0.53369 (2)	0.24668 (4)	0.03643 (14)	
O1	0.2600 (2)	0.03105 (12)	0.0185 (3)	0.0546 (5)	
O2	0.04285 (16)	0.36076 (15)	−0.1653 (3)	0.0568 (4)	
O3	0.2120 (4)	0.7950 (3)	0.9081 (7)	0.1392 (14)	
H3	0.227 (5)	0.8648 (16)	0.931 (7)	0.135 (6)*	
N1	0.44301 (17)	0.37995 (14)	0.1875 (3)	0.0365 (4)	
N2	0.46418 (17)	0.18588 (15)	0.1998 (3)	0.0367 (4)	
N3	0.15129 (17)	0.19704 (15)	−0.0707 (3)	0.0399 (4)	
N4	0.23455 (17)	0.38436 (14)	0.0017 (2)	0.0381 (4)	
C1	0.51491 (19)	0.28771 (17)	0.2480 (3)	0.0368 (5)	
H1	0.5935	0.2955	0.3177	0.044*	
C2	0.35017 (19)	0.21287 (15)	0.0998 (3)	0.0329 (4)	
C3	0.2555 (2)	0.13741 (16)	0.0164 (3)	0.0377 (5)	
C4	0.1381 (2)	0.31716 (19)	−0.0830 (3)	0.0393 (5)	
C5	0.33876 (18)	0.33133 (15)	0.0931 (3)	0.0318 (4)	
C6	0.0440 (2)	0.1291 (2)	−0.1542 (4)	0.0554 (6)	
H6A	−0.0174	0.1442	−0.0819	0.083*	
H6B	0.0635	0.0481	−0.1466	0.083*	
H6C	0.0157	0.1507	−0.2887	0.083*	
C7	0.2229 (3)	0.5112 (2)	−0.0110 (4)	0.0520 (6)	
H7A	0.2696	0.5456	0.1054	0.078*	
H7B	0.1402	0.5323	−0.0236	0.078*	
H7C	0.2513	0.5384	−0.1232	0.078*	

### Atomic displacement parameters (Å^2^)

**Table:**

	*U*^11^	*U*^22^	*U*^33^	*U*^12^	*U*^13^	*U*^23^
Ni1	0.0411 (2)	0.01831 (18)	0.0477 (2)	−0.00491 (9)	0.00408 (13)	−0.00290 (8)
O1	0.0644 (13)	0.0267 (8)	0.0696 (11)	−0.0096 (7)	0.0068 (10)	−0.0035 (6)
O2	0.0416 (9)	0.0613 (11)	0.0608 (10)	0.0094 (8)	−0.0050 (8)	0.0053 (8)
O3	0.118 (3)	0.0735 (17)	0.203 (4)	−0.0072 (19)	−0.019 (3)	0.001 (2)
N1	0.0405 (10)	0.0246 (7)	0.0428 (9)	−0.0015 (7)	0.0052 (8)	−0.0014 (7)
N2	0.0397 (10)	0.0244 (8)	0.0437 (9)	0.0021 (7)	0.0036 (8)	0.0010 (7)
N3	0.0380 (10)	0.0387 (9)	0.0406 (9)	−0.0066 (8)	0.0027 (8)	−0.0010 (7)
N4	0.0413 (10)	0.0291 (8)	0.0419 (9)	0.0054 (7)	0.0040 (8)	0.0035 (7)
C1	0.0370 (12)	0.0269 (12)	0.0438 (12)	0.0005 (8)	0.0022 (10)	0.0003 (7)
C2	0.0375 (11)	0.0240 (9)	0.0363 (10)	−0.0006 (8)	0.0055 (8)	−0.0002 (7)
C3	0.0473 (13)	0.0286 (10)	0.0381 (10)	−0.0049 (8)	0.0107 (9)	−0.0007 (7)
C4	0.0409 (12)	0.0409 (11)	0.0358 (10)	0.0024 (9)	0.0067 (9)	0.0003 (9)
C5	0.0383 (11)	0.0230 (8)	0.0339 (9)	0.0022 (8)	0.0072 (8)	0.0006 (7)
C6	0.0480 (14)	0.0587 (15)	0.0562 (14)	−0.0190 (12)	0.0033 (12)	−0.0054 (11)
C7	0.0574 (16)	0.0295 (10)	0.0639 (15)	0.0110 (11)	0.0008 (12)	0.0038 (10)

### Geometric parameters (Å, °)

**Table:**

Ni1—N2^i^	1.8577 (17)	N4—C5	1.370 (3)
Ni1—N1	1.8636 (17)	N4—C4	1.374 (3)
O1—C3	1.228 (2)	N4—C7	1.469 (3)
O2—C4	1.226 (3)	C1—H1	0.9300
O3—H3	0.832 (11)	C2—C5	1.372 (3)
N1—C5	1.355 (3)	C2—C3	1.414 (3)
N1—C1	1.356 (3)	C6—H6A	0.9600
N2—C1	1.321 (3)	C6—H6B	0.9600
N2—C2	1.377 (3)	C6—H6C	0.9600
N2—Ni1^ii^	1.8577 (17)	C7—H7A	0.9600
N3—C4	1.394 (3)	C7—H7B	0.9600
N3—C3	1.398 (3)	C7—H7C	0.9600
N3—C6	1.467 (3)		
			
N2^i^—Ni1—N1	177.25 (8)	O1—C3—C2	125.7 (2)
C5—N1—C1	103.84 (16)	N3—C3—C2	112.53 (17)
C5—N1—Ni1	131.82 (14)	O2—C4—N4	121.4 (2)
C1—N1—Ni1	124.22 (14)	O2—C4—N3	120.7 (2)
C1—N2—C2	104.20 (15)	N4—C4—N3	117.9 (2)
C1—N2—Ni1^ii^	133.63 (15)	N1—C5—N4	129.03 (17)
C2—N2—Ni1^ii^	122.05 (14)	N1—C5—C2	109.16 (18)
C4—N3—C3	125.93 (19)	N4—C5—C2	121.81 (19)
C4—N3—C6	115.8 (2)	N3—C6—H6A	109.5
C3—N3—C6	118.24 (19)	N3—C6—H6B	109.5
C5—N4—C4	119.16 (17)	H6A—C6—H6B	109.5
C5—N4—C7	122.08 (19)	N3—C6—H6C	109.5
C4—N4—C7	118.75 (19)	H6A—C6—H6C	109.5
N2—C1—N1	114.44 (18)	H6B—C6—H6C	109.5
N2—C1—H1	122.8	N4—C7—H7A	109.5
N1—C1—H1	122.8	N4—C7—H7B	109.5
C5—C2—N2	108.36 (18)	H7A—C7—H7B	109.5
C5—C2—C3	122.7 (2)	N4—C7—H7C	109.5
N2—C2—C3	128.95 (17)	H7A—C7—H7C	109.5
O1—C3—N3	121.7 (2)	H7B—C7—H7C	109.5

Symmetry codes: (i) −*x*+1, *y*+1/2, −*z*+1/2; (ii) −*x*+1, *y*−1/2, −*z*+1/2.

### Hydrogen-bond geometry (Å, °)

**Table:**

*D*—H···*A*	*D*—H	H···*A*	*D*···*A*	*D*—H···*A*
O3—H3···O1^iii^	0.832 (11)	2.023 (12)	2.851 (3)	173 (5)

Symmetry codes: (iii) *x*, *y*+1, *z*+1.
